# Morpho-molecular, cultural and pathological characterization of *Athelia rolfsii* causing southern blight disease on common bean

**DOI:** 10.1016/j.heliyon.2023.e16136

**Published:** 2023-05-17

**Authors:** Swapan Kumar Paul, Dipali Rani Gupta, Chandan Kumar Mahapatra, Kanistha Rani, Tofazzal Islam

**Affiliations:** a Department of Agronomy, Bangladesh Agricultural University, Mymensingh, 2202, Bangladesh; b Institute of Biotechnology and Genetic Engineering, Bangabandhu Sheikh Mujibur Rahman Agricultural University, Gazipur, 1706, Bangladesh

**Keywords:** Fungal morphology, ITS, Phylogeny, Pathogenicity, Host range

## Abstract

Common bean (*Phaseolus vulgaris*), is a winter legume crop in Bangladesh and is considered an important vegetable with export potential. However, the production of common bean is severely affected by a newly reported soilborne fungal pathogen, *Athelia rolfsii*. This study aimed to characterize this new pathogen by morphological, molecular, cultural, and pathological analyses and determine the host range. The disease incidence in the affected field ranged between 6 and 13%. Initial disease symptoms were observed as brown sunken lesions at the point of infection and development of mycelia, followed by yellowing and quick wilting of the whole plant. A total of 10 fungal isolates were recovered from the infected plant samples, which were morphologically similar and produced white to brown mycelia and numerous brown sclerotia on the PDA medium. Two of them viz. BTCBSr3 and BTCBSr4 were used for the detailed study. Based on morphology and phylogenetic analyses of the sequenced data of internal transcribed spacer (ITS) and translation elongation factor 1 alpha (EF-1α), the pathogen was identified as *A. rolfsii*. Mycelial growth rate (3.6 cm/day) and fresh weight (107 mg) were higher in the PDA medium, whereas the number of sclerotia production (328/plate) was higher in OMA media. The isolates could grow in a wider range of incubation temperatures (15–35 °C) and media pH (3–9). In the cross-inoculation assay, both isolates were pathogenic on tomato, brinjal, and chickpea, but not on chili, soybean, and cowpea. This study has laid a foundation for further pathological research on the fungus in aid to develop an effective management practice against the pathogen.

## Introduction

The soilborne fungal pathogen *Athelia rolfsii* (Curzi) Tu & Kimbrough [anamorph, *Sclerotium rolfsii* Sacc.] is a member of basidiomycetes and known to infect more than 500 plant species [1]. The pathogen causes southern blight or root rot disease in different crops which results in significant yield losses worldwide [[2], [3], [4], [5]]. The disease was first identified on tomato plants and later reported on many other crops including common bean, soybean, potato, cowpea, lentil, sesame, chili and brinjal [[5], [6], [7], [8], [9], [10]]. Although yield loss by the pathogen attack depends on the crop species but warm and moist climates create a suitable microenvironment for successful disease establishment for the pathogen [11,12]. The pathogen overwinters and survives as sclerotia in soil and on diseased plants or residues for a long time [[13], [14], [15], [16]]. Under favorable conditions, it produces mycelia from overwintered inoculum and can cause infection when a new crop is grown the following season [17]. When the mycelia come to contact of host plants, the pathogen infects the stem or root near the soil. In common beans, the first visible symptom appears as water-soaked lesions or brown discoloration on the stem above the soil. Later yellowing of leaves and rotten stem and root along with white mycelia become evident [5,18]. *A. rolfsii* produces abundant white mycelia and brown to black sclerotia on stem, root, and soil near the root surface of the host plants [19,20]. Mycelial growth and sclerotia formation of *A. rolfsii* is affected by several factors among which temperature, and soil pH are two important factors [[20], [21], [22], [23], [24]]. The pathogen can grow in a wide range of temperature variations but a temperature ranges between 25 and 30 °C is optimum for mycelial growth and sclerotium production [14,20,25]. The optimum pH range for mycelial growth is 4–8 [25]. In addition, soil water content also affects mycelial growth, sclerotial formation, and disease development [24].

Identification of *Sclerotium* at the genus level is traditionally reliant on cultural and morphological descriptions. However, these identification techniques are limited to the delimitation of *Sclerotium* to the species level [26]. Although *A.* *rolfsii* is differentiated from its two other closely related species *S. delphinii* and *S. coffeicola* by sclerotial morphology and growth sometimes they produce indistinguishable morphology at high temperatures [[26], [27], [28]]. Moreover, *A. rolfsii* isolates from different hosts and geographical zones frequently exhibit variable morphological characteristics when cultured in the medium [5,29,30]. While species delimitation using morphology remains insufficient for the resolution of *Sclerotium* at the species level, sequencing of the conserved regions or genes such as *ITS*- region, EF-1α gene and phylogenetic analyses based on the sequences have proven reliable in addressing challenges in the identification of *Sclerotium* [30,31]. Accurate identification of pathogens both morphological and molecular analysis is vital for the development of effective management strategies to control the disease.

Common bean, also known as French bean or bush bean, is one of the important legumes used as green vegetables or pulses worldwide [32]. It is an important source of protein, zinc, iron, fiber and antioxidants [33,34]. It reduces the demand for nitrogen fertilizer by biological nitrogen fixation in the soil. In Bangladesh common bean is widely cultivated in the northern and eastern parts of the country mainly in the winter season [35]. The crop has gained popularity among growers for its short duration and high production rate. However, the production of common bean is severely hampered by a number of diseases, among which southern blight, caused by *A. rolfsii*, is the destructive one. Previous work in Bangladesh reported that common bean yield loss is caused by *A. rolfsii* infection in an experimental plot was ranged from 55.43 to 64.94% by artificial inoculation [35]. However, despite the threat of yield loss, knowledge of pathogen morphology, pathology and factor affecting the growth of the pathogen are lacking. Moreover, molecular identification of the pathogen has not been done. Therefore, the objectives of this study were to, first, identify the pathogenic fungus of southern blight disease on common beans by morphological, pathological, and molecular analysis; second, determine the optimum conditions for growth and sclerotia formation of the pathogen; and third, asses the host-range of the selected isolates of this fungal pathogen.

## 2 Materials and methods

### 2.1 Disease incidence, collection and isolation of *A. rolfsii* isolates

In January 2022, disease incidence of southern blight of common bean plants was monitored in an experimental field of Bangladesh Agricultural University (24.75° N, 90.50° E), Mymensingh and Percent Disease Incidence was calculated according to the methods described by Le et al. [36]. A total of 5 infected plant samples having the typical southern blight symptoms were collected from randomly selected 5 experimental plots by uprooting the plants. For isolation of *A. rolfsii*, infected plants exhibited white mycelia on stem surface were surface sterilized with 1% NaOCl followed by washing with sterilized water and blotted dry between sterile filter papers. Infected stem (5 mm × 5 mm) was cut from the advancing disease area and placed on PDA medium and incubated at 25 °C for 3 days in the dark for growth of mycelia. Fungal isolates were purified by repeated tip culture. At least two isolates were recovered from each sample with a total of ten fungal isolates. As the isolates showed similar morphological characteristics, two randomly selected isolates were used in this study.

### 2.2 Morphological characterization

Morphological characterizations were done by growing the *A. rolfsii* isolates on PDA medium at 25 °C in the dark. Approximately, 3 mm mycelial block from growing fungal culture were cut and transferred onto Petri dish plate containing PDA medium. Plates were incubated at 25 °C and radial growth of colonies was measured by a ruler in every 24 h interval for 3 days. Plates were incubated for 20 days at 25 °C to monitor the sclerotia formation. The numbers of sclerotia produced by the isolates were counted manually and size was measured by a vernier caliper. The size of mycelia and, production of clamp connections on mycelia was observed under a microscope and photographs were taken by a camera attached with it (CarlZeiss, Gottingen, Germany). Each of the experiment was repeated twice.

### 2.3 Molecular characterization

The genomic DNA of the fungal isolates was extracted by scrapping the mycelia from 4 days old culture using the Wizard Genomic DNA Purification Kit (Promega, USA) according to the manufacturer's instructions. The PCR reactions were carried out using primers ITS1/ITS4 and EF595F/EF1160R to amplify the internal transcribed spacer region (ITS region) and translation elongation factor 1 alpha (EF-1α) gene, respectively [37,38]. The PCR mixture contained 0.1 μl of DNA Taq polymerase (2.5 U), 5 μl of 10 × polymerase buffer, 3 μl of 25 mM MgCl2, 1 μl of 10 mM dNTP, 2 μl of 20 pmol/μl of each primer, and 1 μl of the template (extracted genomic DNA at 50 ng/μl) in a total volume of 50 μl (Promega, WI, USA). The thermal program for the ITS region consisted of maintaining a temperature at 95 °C for 2 min, followed by 95 °C for 40 s, 55 °C for 40 s, 72 °C for 60 s and a final extension of 5 min at 72 °C. For amplification of EF1-α gene, the PCR conditions were 95 °C for 2 min, followed by 95 °C for 40 s, 54 °C for 35 s, 72 °C for 60 s, and a final extension of 5 min at 72 °C. The PCR-amplified fragments were observed in a UV light transilluminator. The amplified products were purified and sent for sequencing (Apical Scientific Sequencing, Malaysia). The obtained nucleotide sequences were then aligned with other ITS and EF-1α gene sequences of several *Athelia* reference species retrieved from the NCBI GenBank using the BLAST algorithm. The nucleotide sequences were analyzed and highly homologous sequences were aligned using Clustal-X version 2.0.11. The newly obtained sequences were submitted to NCBI GenBank for accession number. Maximum likelihood analysis was performed using the MEGA7 software with a bootstrap test with 1000 replicates [39].

### 2.4 Inoculum preparation and pathogenicity test

*Athelia rolfsii* inoculum was prepared following the method described by Borkar & Gawande [40] with some modifications. Briefly, 100 g of moistened wheat seeds were autoclaved at 120 °C for 15 min in an Erlenmeyer flask. Approximately, 50 mm of agar blocks were cut from 3 days old *A. rolfsii* culture and placed in the flask. The flask was then incubated for 15 days at 25 °C and with a 12 h photoperiod. Two isolates of *A. rolfsii* namely, BTCBSr3 and BTCBSr4 were used for inoculum preparation. The infested wheat seeds were used as inoculum of *A. rolfsii*. For the pathogenicity test, seedlings (cv. BARRI Jharshim-3) were raised in pots containing a sterile potting mixture (field soil/composted manure/sand 2:2:1 [v/v]). Twenty days old plants were inoculated by placing three infested wheat seeds adjacent to the collar region. Un-inoculated autoclaved wheat grains served as control. Plants were placed in a growth room with a 16 h/8 h light/dark photoperiod at 25 ± 2 °C after inoculation. Developments of symptoms were monitored every three days’ intervals. Un-inoculated autoclaved wheat grains served as control. To confirm the presence of the pathogen, fungi were reisolated from artificially inoculated common bean roots and cultured on PDA plate. To test the host range of *A. rolfsii* isolates BTCBSr3 and BTCBSr4, some commonly grown winter vegetables such as tomato (cv. BARI Tomato-9), chili (cv. BARI Morich-1), brinjal (cv. BARI Brinjal-8), soybean (cv. BARI Soybean-5), cowpea (cv. BARI Felon-1), and chick pea (cv. BARI Chola-4) were selected for cross inoculation. All the seedlings were raised in the plastic pots containing potting mix and 15–20 days old seedlings were subjected to A. *rolfsii* inoculation following the method as described earlier.

### *2.5* Effect of culture media, temperature and pH on growth and sclerotia production of *A. rolfsii* isolates

All the media used in this study were prepared following the manufacturer's protocol. To test the effects of culture media on growth, biomass production, and sclerotia formation *A. rolfsii* isolates were grown on PDA, CMA, and OMA media. A plug of 5 mm diameter mycelia was cut from the developing colony of *A. rolfsii* and placed on the respective media. The plates were then incubated at 25 °C in the dark and the radial growth of the isolates was measured every 24 h at intervals for 3 days using a scale. Total mycelia were harvested by scrapping from the 3-day-old culture plates and weighed in a balance. The effect of temperatures and pH on radial growth, mycelial fresh weight and sclerotia production was also monitored by growing the isolate on PDA at diverse temperatures and pH ranges. Mycelium plugs of 5 mm diameter were cut from a 3-day-old culture and placed on PDA plates and incubated at 15, 20, 25, 30, and 40 °C in the dark. Similarly, PDA media were prepared with a pH of 3.0, 5.0, 7.0, and 9.0 and isolates were cultured as described above. The cultures were incubated at 25 °C in dark with six replicates for each treatment. The radius of colonies was measured at 24, 48, and 72 h after incubation. Cultures were monitored for sclerotial development and then the number of sclerotia were counted after 20 days of incubation. The experiment was repeated twice.

### 2.6 Statistical analyses

The experiment was laid out in a Completely Randomized design (CRD). Obtained data were subjected to one-way analysis of variance (ANOVA) and means were separated using Fisher's protected LSD (at p ≤ 0.05) using SPSS version 16 and Microsoft Office Excel 2010 program packages for preparing graphs.

## 3 Results

### 3.1 Disease symptoms and incidence

**Fig. 1:**
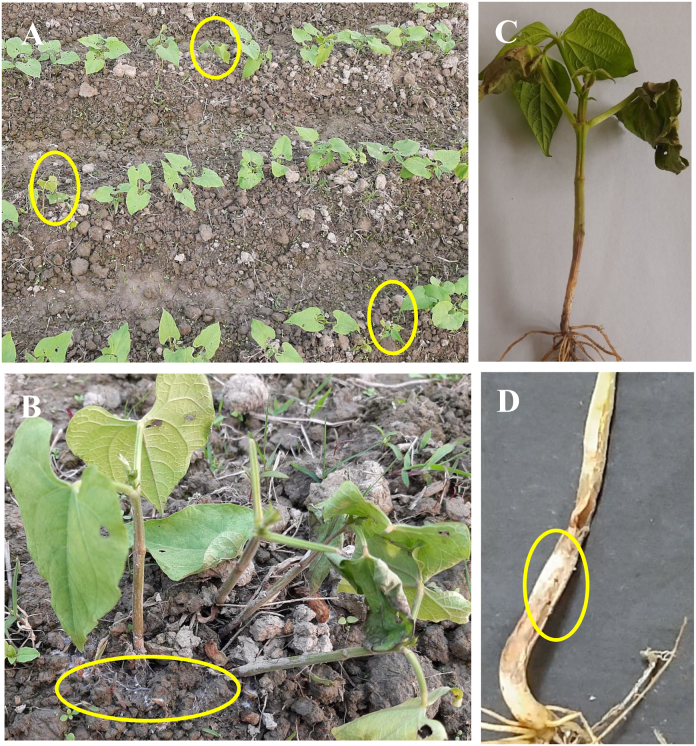
Southern blight disease symptoms; A. Naturally infected common bean field (yellow circles indicate the infected plat in field); B. Presence of mycelial mat on soil surface (yellow circle indicates the mycelial mat); C. Infected common bean plant; D. Infected stem with mycelia (marked in yellow circle).

Disease symptoms are mainly observed at the base of the main stem of the common bean plants (Fig. 1). Brown sunken lesions appeared at the collar region near the soil of the infected plants. The leaves became wilted and dried from the bottom of the plants to the top (Fig. 1B). Cotton-white mycelium appeared at the base of the stems near the soil surface (Fig. 1B). Tissues became soft and rotten at the portion of infection leading to the complete death of plants (Fig. 1D). An estimated 6–13% disease incidence was recorded in the field.

### 3.2 Morphological characterization

**Fig. 2:**
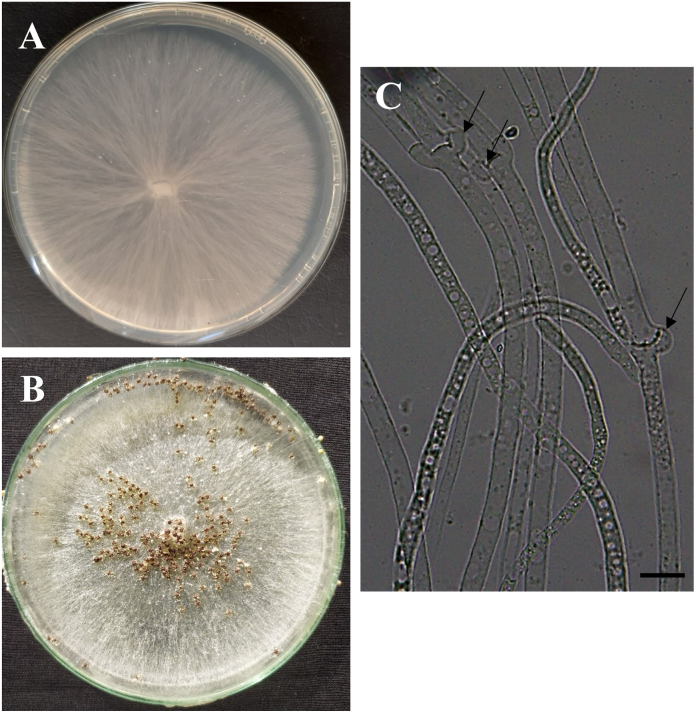
Macroscopic and microscopic view of *S. rolfsii* isolate BTCBSr3; A. Colony morphology of isolates BTCBSr3 on PDA; B. Production of sclerotia; C. Microscopic view of mycelia and clamp connection. Scale bar = 5 μm.

Two isolates namely, BTCBSr3 and BTCBSr4 were used for morphological study. Both the isolates had the same morphology on the PDA medium. Isolates produced white cottony and compact mycelia on PDA having a growth rate 3.6 cm/day (Fig. 2A). Microscopic observation showed that the mycelium was hyaline and branched with septate hyphae. Mycelium was 3–9.4 μm in diameter and presence of a clamp connection (Fig. 1, Fig. 2C). Abundant sclerotia were produced on PDA plates which were white during the early stage of growth but turned brown at a later stage. Sclerotia were round, spherical, or irregularly elliptical, with a smooth, shiny surface, and measured 0.5–1.1 mm in diameter (Fig. 2B).

### 3.2 Molecular identification of the isolates

**Fig. 3:**
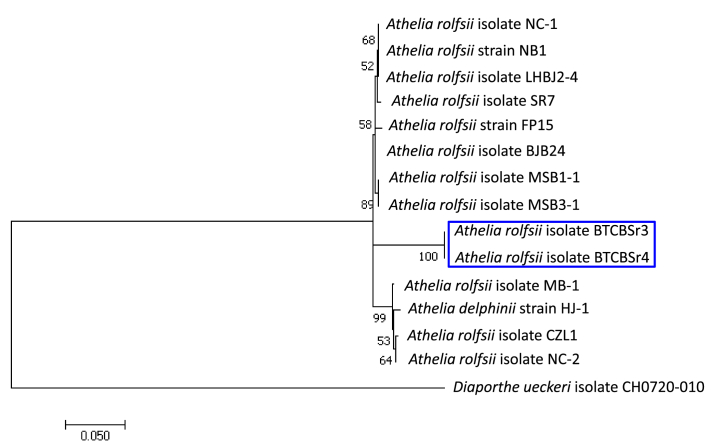
Phylogenetic tree based on the sequences of ITS region and EF-1α gene from *A. rolfsii* isolates BTCBSr3 and BTCBSr4 along with other *A. rolfsii* isolates using the Maximum Likelihood method and Tamura 3-parameter model. Isolates of this study are given in a blue color rectangle.

The sequences of ITS region of the isolates BTCBSr3 (ON195575.1) and BTCBSr4 (ON207520.1) showed 98.14% homology with *A. rolfsii* strain cpsr (KC894860.1) and EF-1α of the isolates BTCBSr3 (OQ732628) and BTCBSr4 (OQ732629) showed 99.80% sequence homology with *A. rolfsii* isolate BJB24 (MF375218.1) from GenBank by BLAST search analysis. Phylogenetic analysis using ITS and EF-1α revealed that both the isolates grouped with other *A. rolfsii* isolates with a good bootstrap value (Fig. 3).

### 3.3 Pathogenicity test

**Fig. 4:**
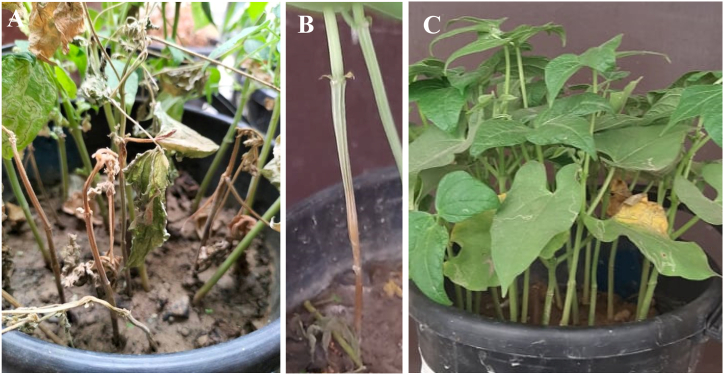
Pathogenicity assay of *A. rolfsii* isolates BTCBSr3 on common bean plants; A. Infected plants inoculated with the isolate BTCBSr3; B. Disease symptom on common bean stem; C. Healthy control plants.

Southern blight symptoms appeared 7–10 days after inoculation of *A. rolfsii* isolates in common bean plants. Initially, brown discoloration appeared at the base of the stem and the plants become wilted and stem become dried during the disease progression stage. Abundant white cottony aerial mycelium was observed in the stem near the soil and soil surface, which were similar to those observed in the field (Fig. 4A and B). The control plants remained healthy (Fig. 4C). Fungus was reisolated from the infected tissues of the common bean plants and characterized by morphological and molecular identification. These results indicate that the isolates BTCBSr3 and BTCBSr4 are the causal agent of the southern blight disease of common bean.

### 3.4 Effects of culture media on growth, mycelial fresh weight and sclerotia production of *A. rolfsii*

**Fig. 5:**
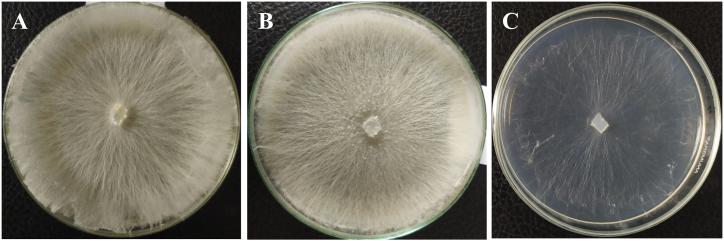
Effects of culture media on radial growth rate, mycelial fresh weight and sclerotial initiation of the isolate BTCBSr3; A. PDA media; B. OMA media; C. CMA media.

Both isolates grew well in all three media tested. Since no significant difference was observed in regard to their growth rate, mycelial fresh weight, and sclerotia production, we presented the mean data of the isolates BTCBSr3. The radial growth rate was higher in PDA (3.6 cm/day) followed by OMA (3.41 cm/day) and CMA (2.61 cm/day) (Table 1). In PDA and OMA, isolates produced cottony white and compact mycelia whereas in CMA isolates produced sparse and less densely mycelia (Fig. 5A–C). Mycelial fresh weight was significantly (at p ≤ 0.05) affected by the culture media. Higher mycelial fresh weight was recorded in PDA (107.17 mg) media compared to OMA (49.50 mg) and CMA (11.43 mg). Interestingly, a higher amount of sclerotia production was recorded in OMA (328.33/plate) followed by PDA (268.33/plate) and CMA (21.33/plate) media. Media also affected the sclerotia initiation. In OMA, the sclerotia initiation started on 3–4th days of incubation whereas in PDA and CMA sclerotia initiation started on 7–9th and 7–10th days of incubation, respectively (Table 1).

**Table 1:** Effects of culture media on radial growth rate, mycelial fresh weight and sclerotial initiation and production of the isolate BTCBSr3.

Media	Radial growth (cm)^a^	Mycelial fresh weight (mg)^a^	Days of sclerotia initiation	Sclerotia production (no./plate)^a^
PDA	3.6 ± 0.15^a^	107.17 ± 1.28^a^	7–9	268.33 ± 12.14^b^
OMA	3.41 ± 0.07^a^	49.50 ± 1.95^b^	3–4	328.33 ± 8.51^a^
CMA	2.61 ± 0.09^b^	11.43 ± 0.97^c^	7–10	21.33 ± 6.50^c^

a Values are Mean ± SE. Mean values of each column with the same letter(s) do not differ significantly by LSD (P ≤ 0.05).

### 3.5 Effects of temperatures on mycelial growth, mycelial fresh weight and sclerotia production of *A. rolfsii*

**Fig. 6:**
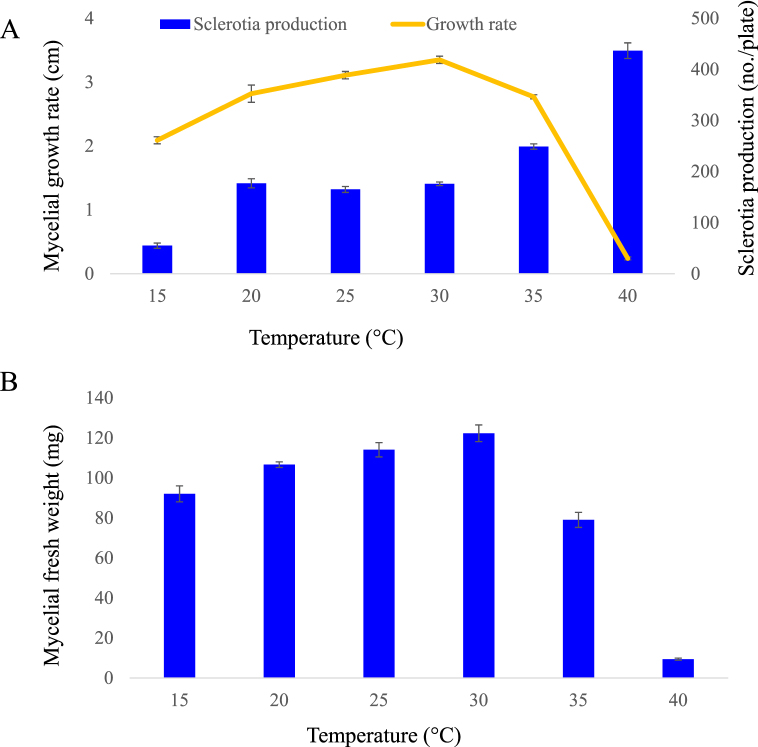
Effect of temperatures on radial growth rate, mycelial fresh weight and sclerotia production; A. Linear and bar graphs represent the mean radial growth rate and sclerotia production, respectively; B. Bar graph represent the mycelial fresh weight of the isolates BTCBSr3 on PDA. Bars above the column indicate standard error of the mean.

Mycelial radial growth was significantly (at p ≤ 0.05) affected by the incubation temperature. As shown in Fig. 6A, the isolate was able to grow over a range of temperatures varying from 15 °C to 35 °C, whereas very little growth was observed at 40 °C. In general, the rate of mycelial growth increased as temperature increased up to 30 °C and then decreased rapidly as temperature increased. Mycelial growth was slightly slowed at 15 °C with an average rate of 2.09 cm/day, compared to the growth rates of 2.82, 3.11, 3.35, and 2.77 cm/day, noted at 20 °C, 25 °C 30 °C, and 35 °C, respectively. Mycelial fresh weight varied significantly at different incubation temperatures. The optimum mycelial fresh weight ranged from 106.64 to 122.33 and was recorded between the temperature range 20–30 °C. However, the lowest was recorded at 40 °C which was 9.42 mg (Fig. 6B). Temperature also affects the sclerotia production in *A. rofsii*. Ther sclerotial formation was delayed at 15 °C and no mature sclerotia were produced even after 20 days of incubation. An early and maximum number of sclerotia (an average of 437/plate) production was noted at 40 °C. Isolate produced an average of 177, 165, 176, and 249 sclerotia/plate at 20, 25, 30 and 35 °C, respectively (Fig. 6A).

### 3.6 Effects of pH on mycelial growth, mycelial fresh weight and sclerotia production of *A rolfsii*

**Fig. 7:**
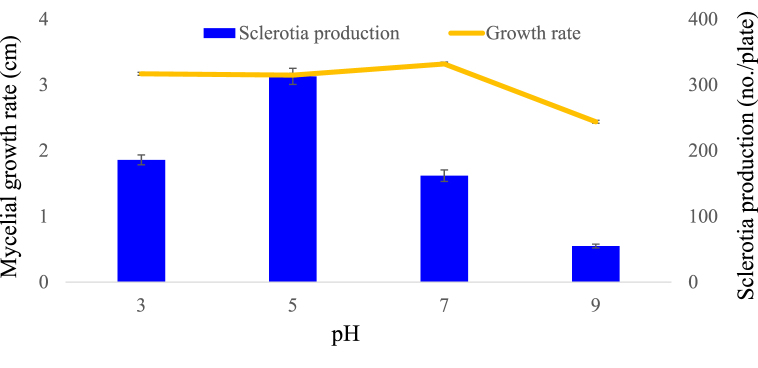
Effect of different pH on radial growth rate and sclerotia production of the isolates BTCBSr3 on PDA. Linear and bar graphs represent the mean radial growth rate and sclerotia production, respectively. Bars above the column indicate standard error of the mean.

The mycelial growth rate was significantly affected by the tested media pH. Although the isolates grew well in a wide range of pH ranging from 3 to 9, but the growth was slightly slowed at pH 9 (Fig. 7). Optimum mycelial growth was recorded at pH 7 (3.32 cm/day) followed by pH 3 (3.17 cm/day), pH 5 (3.15 cm/day), and pH 9 (2.44 cm/day). Mycelial fresh weight was not significantly affected by the media pH except for 9 (data not shown). However, sclerotia formation was greatly affected by the media pH. Both the isolates showed the maximum number of sclerotia production at pH 5 (313) followed by pH 3 (186), pH 7 (162), and pH 9 (55).

### 3.7 Host range of *A. rolfsii* isolates

**Fig. 8:**
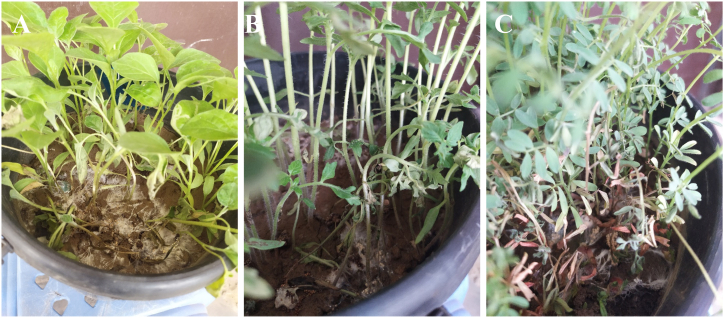
Host range of the isolates BTCBSr3; A. Development of disease symptom on brinjal; B. Tomato; C. Chick pea. Plants were inoculated with *A. rolfsii* isolate BTCBSr3 infected wheat seeds and development of disease symptoms was monitored in every three days interval.

Both the isolates were found to be highly pathogenic on tomatoes, brinjal, and chickpea. Isolates produced symptoms on the collar and stem of the plants. Tomato and eggplant became collapsed within 7 days after pathogen inoculation (Fig. 8A and B). On chickpeas, the initial symptom appeared at 12 days after pathogen inoculation. At first brown discoloration was observed on the stem and gradually whole plants became yellow and dried (Fig. 8C). However, the isolates did not produce any symptoms on chili, soybean, and cowpea (data not shown).

## 4 Discussion

In the current study, we demonstrated the morphological, molecular, and pathological features of *A. rolfsii,* a causal fungal pathogen of the southern blight disease of common bean in Bangladesh. Although the severity of crop yield losses by the infection of *A. rolfsii* has recently been reported by Alam et al. [35], this study for the first time reported the morphological, molecular and pathological characterization of this destructive fungus causing southern blight disease of common bean in Bangladesh. Furthermore, this study also determined the factors influencing the growth and sclerotia formation and host range of *A. rolfsii* isolated from the common beans in Bangladesh to facilitate the easy and rapid production of inocula for large-scale germplasms screening and development management practices.

The fungus *A. rolfsii* is a soilborne pathogen attacking more than 500 plant hosts in the world [1]. They generally caused disease on root, collar, and stem near the soil in warmer and humid conditions. In our study, the fungus was associated with the southern blight disease of the common bean. The isolates recovered from the symptomatic tissue produced pale brown to white cottony mycelia having a faster growth rate (3.6 cm/day) on PDA media. Mycelia were hyaline, thin walls, septate and possessed clamp connections (Fig. 2). The isolates also produced abundant brown sclerotia with 0.5–1.1 mm diameter. Based on the morphology, the pathogen was primarily identified as *A. rolfsii* [19,41]. The fungus *S. rolfsii* has been reported to generally produce white fluffy or compact type mycelia [36,42]. Some isolates from silky-white mycelia, while some of them produce white to pale-brown mycelia have been reported [20,30,36]. Variations among the sclerotia production and their size and shape have also been reported by many earlier investigators [5,28,30]. Members of the genus *Sclerotium* produce only sclerotia and no fruiting bodies or spores, therefore, difficult identify the species by only morphological characterization [31]. Molecular characterization by sequencing conserved DNA regions is a powerful tool for the correct characterization of pathogens at species level [43]. The ITS and EF1-α sequencing and phylogenetic analysis of the present isolates revealed that both the isolates grouped with *A. rolfsii* isolates with a good bootstrap value and showed 98.14 and 98.80% sequence similarity with *A. rolfsii* isolate cpsr of cowpea and BJB24 of *Aconitum carmichaelii*. Xu et al. [31] determined the phylogenetic placement of eight *Sclerotium* spp. by analyzing the ITS and LSU sequence. Molecular characterization of *A. rolfsii* by ITS and EF1-α sequencing has previously been reported in many other crops [12,20,[44], [45], [46]].

In our study, we found that PDA was suitable for the mycelial growth and fresh weight of the pathogen. The suitability of PDA medium for mycelial growth of *A. rolfsii* has previously been reported [20,22,30]. Here, we reported the OMA media is also good for the radial growth of *A. rolfsii* isolates BTCBSr3 and BTCBSr4. However, the mycelial fresh weight was significantly higher in PDA (107.17 mg) media compared to OMA (49.50 mg) and CMA (11.43 mg) media (Table 1). Many earlier researchers reported that most of the fungal isolates thrives well in PDA media and encourage mycelial growth as it is rich in nutrient [47]. Although all three media used in this study were starch-based media the difference in the radial growth and mycelial weight might be attributed to the specific nutrient constituents of the media as well as the availability of nutrients to the pathogen [47,48]. Besides the growth media, the radial growth of *A. rolfsii* was also affected by some other factors such as temperature and pH. In our investigation, we found that the pathogen can grow in a wide range of temperatures. The growth was delayed at 40 °C but it can fairly grow at 20–35 °C and maximum radial growth was achieved at the temperature 30 °C. However, the growth was slightly slowed at 15 °C (Fig. 6A). These results are in agreement with other studies reporting that rapid mycelial growth occurred at 25–30 °C but decreased significantly below 20 °C and above 35 °C [21,23]. One of the interesting findings of our study is that the mycelial radial growth and fresh weight were not greatly affected by the medium pH. Both the isolates were able to grow a wider pH ranging from 3 to 9 in the media (Fig. 7). However, radial growth was slightly restricted at pH 9. Most of studies showed that maximum radial growth of *A. rolfsii* occurred at pH 6–7 [20,22,25,49,50] and markedly less above pH 8 [1,14].

Production of sclerotia is known to play a vital role in over-winter pathogen in the soil or plant debris. Culture media, temperature, and pH affect the sclerotia initiation and production of *A. rolfsii* isolates BTCBSr3 and BTCBSr4. An early sclerotial initiation occurred in OMA media and started from the 3–4th day of incubation (Fig. 3, Fig. 5and Table 1). Sclerotia initiation started from 7 to 10th days of incubation for both PDA and CMA media. Although on PDA, isolates showed faster radial growth and mycelial fresh weight but were slow to produce sclerotia and formed fewer sclerotia than OMA (Fig. 3, Fig. 5 and Table 1). Variations of sclerotia production in different media have been reported by some other researchers [20,22]. A previous study reported that sclerotia production was delayed in nutrient-rich medium and induced in poor medium [51]. Although the exact nutrient constituent of the used media is not well known our observations suggested that fugal morphogenesis in various media is tightly linked with the availability of nutrients to the pathogen.

Sclerotia production is known to affect by incubation temperatures and pH of the culture medium. Isolates of *A. rolfsii* from common beans produced the maximum number of sclerotia (437/plate) at 40 °C and the lowest at 15 °C (55/plate). These results are in agreement with the results obtained by Paul et al. [20] who noted a higher amount of sclerotia production in *A. rolfsii* isolates of sugar beet at increased temperature. It has been reported that the increase in temperature causes an increase in oxidative stress to the cell by denaturation of enzymes related to the metabolism of molecular oxygen and to hemolytic breakage of some cellular compounds [52]. Like media pH also significantly affected the sclerotia production of *A. rolfsii* isolates. Optimum sclerotia production occurred at pH 5 and was good at pH 3 and 7 but reduced at pH 9. Our findings are in agreement with those of Ayed et al. [22] and Sarker et al. [50] reporting maximum sclerotial formation occurred at pH 5 and less in alkaline pH. In another study, Zape et al. [25] reported that higher sclerotia production occurred at pH 7 but optimum at pH between 5.5 and 7.5. Our study suggests that a slightly acidic pH is suitable for higher sclerotia production than alkaline or neutral pH. An acidic pH has also been shown to induce sclerotia production in *Sclerotinia sclerotiarum* by lowering oxalic acid production [53]. It has been reported that acidic pH favors free radical generation, lipid, and protein peroxidation and differentiation that trigger sclerotial formation in *S. rolfsii* [52]. Moreover, intracellular changes in pH might initiate or coordinate with some signaling pathways responsible for sclerotial biogenesis by inducing oxidative stress in the pathogen [52,53].

The fungus *A. rolfsii* has a wider host range which includes vegetables, legumes, forest cereals, and flowers. In our study, *A. rolfsii* isolates of common bean showed pathogenicity toward brinjal, tomato and chickpeas but not on chili, soybean and cowpea (Fig. 8). This result indicates that the isolates have some specificity toward their hosts. A recent study suggests the presence of formae speciales among the *A. rolfsii* isolates of different hosts [40]. They found that isolates from chickpeas, tomatoes, and wheat had a wider host range and could infects peas, onions, green grams, and groundnut, whereas isolates from bottle gourds could not infect them. Another study also reported that isolates of sugar beet were not pathogenic on chili, soybean, and tomato [20]. These findings indicate that the host selection of *A. rolfsii* probably depends on its formae speciales. Therefore, identification of host range of common bean's *A. rolfsii* isolates in Bangladesh might be helpful for formulating suitable cropping systems to avoid the infection of the pathogen. However, further studies are also needed to shed light on the pathogenic variation of *A. rolfsii* isolates infecting common beans in Bangladesh and the development of a sustainable management strategy against this destructive fungal pathogen.

## Author contribution statement

Tofazzal Islam and Swapan Kumar Paul: Conceived and designed the experiments, Contributed reagents, materials, analysis tools or data.

Swapan Kumar Paul, Dipali Rani Gupta, Chandan Kumar Mahapatra and Kanistha Rani: Performed the experiments, Analyzed and interpreted the data.

Tofazzal Islam, Swapan Kumar Paul and Dipali Rani Gupta: Wrote and revised the paper.

## Data availability statement

Data will be made available on request.

## Additional information

Supplementary content related to this article has been published online at [URL].

## Declaration of competing interest

The authors declare that they have no known competing financial interests or personal relationships that could have appeared to influence the work reported in this paper.

## Abbreviations

ITS, Internal Transcribed Spacer; PDA, Potato Dextrose Agar; OMA, Oat Meal Agar; CMA, Corn Meal Agar.

## Appendix ASupplementary data

Supplimentary file_HELIYON-D-22-26301 Supplimentary file_HELIYON-D-22-26301
