# Supplementary material for: Morpho-molecular, cultural and pathological characterization of Athelia rolfsii causing southern blight disease on common bean
Source: Heliyon. 2023 May 17;9(5):e16136. doi: 10.1016/j.heliyon.2023.e16136 (PMC10208843; doi:10.1016/j.heliyon.2023.e16136)
Supplement: Supplimentary file_HELIYON-D-22-26301 [file mmc1.docx]

| Isolate name | Accession number | |
| --- | --- | --- |
|  | ITS | EF1 |
| *Athelia rolfsii* isolate BTCBSr3 | ON195575.1 | OQ732628 |
| *A. rolfsii* isolate BTCBSr4 | ON207520.1 | OQ732629 |
| *A. rolfsii* isolate LHBJ2-4 | MZ956758.1 | OL365370.1 |
| *A. delphinii* strain HJ-1 | MW049362.1 | MW415934.1 |
| *A. rolfsii* isolate MSB1-1 | MN610007.1 | MN702785.1 |
| *A. rolfsii* isolate MSB3-1 | MN610003.1 | MN702787.1 |
| *A. rolfsii* strain NB1 | MN071107.1 | MN509438.1 |
| *A. rolfsii* isolate NC-1 | MW311079.1 | MW322687.1 |
| *A. rolfsii* strain FP15 | KT750883.1 | KF850528.1 |
| *A. rolfsii* isolate MB-1 | OM946593.1 | OL416131.1 |
| *A. rolfsii* isolate SR7 | KP982853.1 | KP982854.1 |
| *A. rolfsii* isolate CZL1 | MT812692.1 | MT846496.1 |
| *A. rolfsii* isolate NC-2 | MW311080.1 | MW322688.1 |
| *A. rolfsii* isolate BJB24 | MF033903.1 | MF375218.1 |
| *Diaporthe ueckeri* isolate CH0720-010 | OK067377.1 | OK149764.1 |

**Table: List of isolates and their genbank accession numbers used in the phylogeny**
